# Effect of Monosodium Urate Crystal Deposition on Atherosclerotic Carotid Plaques

**DOI:** 10.3390/jcm14020518

**Published:** 2025-01-15

**Authors:** Daina Kashiwazaki, Kunitaka Maruyama, Saori Hamada, Shusuke Yamamoto, Emiko Hori, Naoki Akioka, Kyo Noguchi, Satoshi Kuroda

**Affiliations:** 1 Departments of Neurosurgery, Graduate School of Medicine and Pharmaceutical Sciences, University of Toyama, 2630 Sugitani, Toyama 930-0194, Japan; kmaru31@med.u-toyama.ac.jp (K.M.); saori115@med.u-toyama.ac.jp (S.H.); shuyama@med.u-toyama.ac.jp (S.Y.); emihori@med.u-toyama.ac.jp (E.H.); akioka@med.u-toyama.ac.jp (N.A.); skuroda@med.u-toyama.ac.jp (S.K.); 2 Departments of Radiology, Graduate School of Medicine and Pharmaceutical Sciences, University of Toyama, 2630 Sugitani, Toyama 930-0194, Japan; kyo@med.u-toyama.ac.jp

**Keywords:** uric acid, hyperuricemia, atherosclerosis, carotid endarterectomy, plaque

## Abstract

**Background/Objectives:** The accumulation of uric acid in arteriosclerotic plaques has recently attracted attention. Because the interaction between hyperuricemia and atherosclerosis is complex, the details remain obscure. We aimed to elucidate the clinical effect of monosodium urate monohydrate (MSU) deposition on carotid plaques. **Methods:** This study enrolled 89 patients with carotid plaques. MSU deposits were confirmed using Gomori’s methenamine silver staining of carotid endarterectomy (CEA) specimens. To evaluate the macrophage and microvessel marker counts, we used CD68 and CD31. Plaque composition was investigated in carotid plaques with MSU deposition and inflammation. We also examined the use of dual-energy computed tomography (DECT) and compensated for pathological findings to detect MSU crystal deposition in carotid plaques. **Results:** Of the 89 patients who underwent CEA, 31 (34.8%) had hyperuricemia. Overall, 22 (24.7%) participants had MSU deposits and 67 (75.3%) did not. MSU deposits, CD31-positive microvessels, and CD68-positive cells were observed in shoulder lesions. The number of CD31-positive microvessels and CD68-positive cells was higher in patients with MSU deposits than in those without MSU deposits. Most plaques expressing MSU were plaques with intraplaque hemorrhage. The consistency in MSU deposit identification between histopathology and DECT was poor (kappa = 0.34). **Conclusions:** MSU deposition may be related to the inflammation of carotid plaques.

## 1. Introduction

Stroke is the leading cause of morbidity and mortality worldwide. Carotid artery disease accounts for 15% of all neurovascular events [1]. The management of carotid artery disease primarily focuses on the degree of stenosis as the main factor for assessing stroke etiology, future stroke risk, need for intervention, and surgical risk stratification. However, increasing evidence suggests that structural features, plaque composition, and biological activities such as inflammation within the arterial wall and intraplaque hemorrhage (IPH) may have remarkable therapeutic values [2]. High-risk structural features include IPH, a large lipid-rich necrotic core, the thinning and rupture of the fibrous cap, and ulceration. With advancements in imaging technology, these plaque characteristics can be identified using magnetic resonance (MR) imaging, known as plaque imaging, with most features detectable within 5 min using commercially available coils and sequences utilized in routine clinical practice. High-risk plaque composition represents plaques with IPH. The chronic inflammation of the vessel wall is one of the main mechanisms underlying these unstable plaques. In clinical settings, IPH detection by MRI is an efficient clinical method to predict stroke. IPH is an unstable carotid plaque due to the associated inflammation. The plaque rupture of unstable carotid plaques is a major cause of acute ischemic events. Macrophage-related inflammatory responses and microvessel formation primarily induce unstable plaques; consequently, targeting the inflammation and functional modulation of macrophages is clinically crucial for improving plaque stability and inhibiting progression. However, the trigger of the underlying inflammation remains unclear.

Recently, uric acid accumulation in arteriosclerotic plaques has attracted considerable attention. There is histological evidence of uric acid accumulation in carotid plaques obtained from 23 carotid endarterectomy (CEA) specimens [3]. Recently, Nardi et al. reported uric acid as a potential tissue participant and systemic biomarker in the pathogenesis of carotid atherosclerosis, using CEA specimens. Elevated serum uric acid (SUA) levels increase platelet adhesion and stimulate inflammation [3] and endothelial dysfunction [4]. Uric acid-mediated injury to the cell membrane, triggered by inflammation, may lead to the rupture of vulnerable plaque [5]. However, the interaction between hyperuricemia and atherosclerosis is complex. Based on existing knowledge, we hypothesized that uric acid deposition in carotid plaques may act as a trigger of inflammation and may therefore represent a novel therapeutic target, leading to new treatments in the future.

High-resolution dual-energy computed tomography (DECT) is an imaging technique used to detect monosodium urate monohydrate (MSU) crystal deposits in the peripheral joint tissues of patients with gout [6,7] and asymptomatic hyperuricemia [8]. Several recent studies using DECT have suggested that color-coded images consistent with MSU crystals are present in the coronary vessel wall, aorta, and atherosclerotic plaques of most patients with gout [9,10]. However, it remains unclear whether these findings represent true MSU crystal deposition or artifacts from live imaging. Therefore, we conducted a cross-sectional study to determine the association between SUA levels and MSU deposition in the carotid plaques. The radiological features of carotid plaques with MSU deposition and inflammation were also investigated. We also examined the use of DECT and compensated for pathological findings to detect MSU crystal deposition in carotid plaques in patients with gout and hyperuricemia.

## 2. Materials and Methods

### 2.1. Research Ethics

This cross-sectional study was approved by the Institutional Review Board of our university hospital and involved the analysis of a prospective database of patients treated with CEA/carotid artery stenting (CAS) at our institution. Informed consent was obtained from all the patients or their guardians using the opt-out method. Formal informed consent was not required, in accordance with the ethical standards of the institutional research committees. Instead, the outline of the study was made available to the public on our homepage, and an option for patients to decline inclusion in the research was provided.

### 2.2. Study Design and Population

This was a retrospective, cross-sectional study. The inclusion criterion was patients with carotid plaques who were treated with CEA at our institution between April 2015 and December 2022. In total, 97 patients with 97 lesions were diagnosed with carotid stenosis and underwent CEA during the study period; 8 patients (8 lesions) were excluded because of poorly recorded imaging or missing radiological data. Therefore, 89 patients with 89 carotid plaques were included in this study.

Our treatment strategy primarily recommends the use of CEA and CAS for symptomatic and asymptomatic lesions, respectively. However, a crossover of surgical methods was contemplated for patients at risk of complications associated with these procedures. Antiplatelet and statin therapies were administered to patients who underwent CEA. Dual antiplatelet and statin therapies were administered to patients who underwent CAS. Antiplatelet therapy was continued during the perioperative period.

### 2.3. Demographic Data and Laboratory Data

The following clinical data were collected from the hospital charts: age; sex; hypertension; and family history of cardiovascular disease, dyslipidemia, and diabetes. Data on the actual SUA levels and maximal SUA values were collected. Furthermore, data on uric acid-lowering drug use were collected. In this study, hyperuricemia was defined as an elevated SUA level > 7 mg/dL.

### 2.4. Assessing MSU Deposits in Plaque and Stenosis Degree Using DECT

All patients underwent DECT with and without iodine contrast medium administration using third-generation 192-section dual-source computed tomography SOMATOM Force (Siemens Healthcare GmbH, Forchheim, Germany). These patients underwent a non-contrast DECT scan of their carotid artery, using a dual-source CT scanner with simultaneous tube potentials of 80 kV and 110 kV. Examinations were further performed both before and after the administration of the contrast material, which covered the aortic arch to the arterial circle of Willis. The angiographic phase was obtained by administering 60–80 mL of prewarmed contrast medium. The computed tomography technical parameters were as follows: slice thickness = 0.6 mm; matrix size = 512 × 512; and field of view = 20 cm. The degree of stenosis was evaluated using the North American Symptomatic Carotid Endarterectomy Trial method.

### 2.5. Assessing Plaque Composition Using MR Imaging

All patients underwent MR imaging 2 days prior to CEA/CAS. To characterize the plaques, long-axis and axial images of the carotid artery were obtained from T1 sampling perfection with application-optimized contrast using different flip angle evolutions and time-of-flight (TOF) by targeting the area with the culprit lesion. Plaque composition was evaluated using a 1.5 T MR imaging scanner (Magnetom Vision; Siemens, Erlangen, Germany). The three-dimensional T1-weighted sampling perfection with application-optimized contrast was used and the different flip angle evolution technical parameters were as follows: TR/TE = 500/23 ms; variable flip angle; echo-train length = 32; field of view = 250 mm × 240 mm; voxel size = 1.3 mm × 1.0 mm × 1.0 mm; GRAPPA = 2×; and fat suppression = SPAIR. Three-dimensional TOF MR angiography was also performed through both carotid bifurcations in the axial plane using the following imaging sequences. Three-dimensional TOF imaging: field of view = 220 mm/87.5%; repetition time = 23 ms; echo time = 7.00 ms; and slice thickness = 1.2 mm. T1-weighted imaging: field of view = 200 mm/100%; repetition time = 500 ms; echo time = 11 ms; and slice thickness = 4 mm. A signal intensity of >150% of that of the muscle adjacent to the plaque was considered hyperintense. Hyperintense signals in both the T1-weighted and TOF images were diagnosed as plaque with IPH. Hyperintense signals in the T1-weighted images and isointense signals in the TOF images were considered lipid-rich/necrotic cores (LR/NC). Otherwise, an isointense signal in both the T1-weighted and TOF images was diagnosed as a fibrous plaque.

### 2.6. Immunohistochemistry for Urate Crystal

The CEA specimens were divided in two. One part was used for Gomori’s methenamine silver staining and the other for hematoxylin and eosin (HE) staining and immunohistochemistry.

Gomori’s methenamine silver staining is a classic method for staining urate crystals. The deposited urate crystals were stained according to a modified version of Gomori’s method. In brief, CEA specimens were fixed in absolute alcohol at 4 °C overnight, embedded in paraffin and sectioned into 4–5 µm, deparaffinized with xylene and rinsed thrice with absolute alcohol, stained with preheated working methenamine silver for 30 min at 60 °C, toned with gold chloride, and counterstained with eosin solution. The urates were stained black against a blue background.

Furthermore, the carotid artery segments were fixed in 4% phosphate-buffered formalin for at least 48 h and decalcified in 10% ethylenediaminetetraacetic acid for 72 h. Cross-sectioning was performed at 1 cm intervals, and the resulting sections were embedded in paraffin. Each participant received approximately seven paraffin blocks. All tissue sections were cut at 4 μm thickness, mounted on glass slides, and stained with HE. The histological classification of atherosclerosis using the American Heart Association classification was used to characterize the carotid plaque [11].

In total, 89 CEA specimens were obtained from 89 patients. Decalcification was performed using ethylenediaminetetraacetic acid buffer. Specimens were fixed with 4% formaldehyde and embedded in paraffin. Finally, 4 mm-thick axial slices were obtained. A section with the culprit lesion was selected for immunohistochemistry (IHC) analysis. IHC analysis was used to identify macrophages and intraplaque microvessels in the carotid plaques. To evaluate the macrophage and microvessel marker counts, we used CD68 and CD31. Briefly, each section was treated with CD68 (clone PG-M1; 1:100; DAKO, Glostrup, Denmark) and CD31 (rabbit monoclonal; 1:100 dilution; Abcam, ab28364, Cambridge, UK) primary antibodies for 40 min at 24 °C. Incubation with the EnVision polymer from the DAKO EnVision + Kit (DAKO Cytomation, Glostrup, Denmark) was performed for 60 min. Diaminobenzidine tetrahydrochloride chromogen from the diaminobenzidine tetrahydrochloride substitute kit (DAKO Cytomation) was used. The sections were counterstained with hematoxylin. Carotid plaques were divided according to their anatomical location (shoulder, bottom, or core), as previously described. CD68-positive cells and CD31-positive microvessels were counted using the ImageJ software (version 1.61) cell counter tool (National Institutes of Health, Bethesda, MD, USA). The procedures were performed by a certified neurosurgeon.

### 2.7. Blood Sampling

Data were acquired by collecting blood from the peripheral vein of each patient before CEA. Serum C-reactive protein (CRP) and levels were measured from the obtained blood samples as markers of an inflammatory response.

### 2.8. Statistical Analyses

Continuous data are expressed as the means ± standard deviations. Data between subgroups were compared using the Mann–Whitney U test and Fisher’s exact test, as appropriate; the latter was applied if the count in a group was ≤5. The threshold for calcification thickness between symptomatic and asymptomatic carotid plaques was calculated using receiver operating characteristic analysis. Statistical significance was set at *p* < 0.05. Multivariate analyses were performed to compare the data between the thin calcification group and the group without thin calcification. We examined the diagnostic concordance between two methods (DECT and IHC) using inter-rater agreement evaluation (kappa). Statistical analyses were performed using GraphPad Prism version 9.1.0 (GraphPad Software, San Diego, CA, USA).

## 3. Results

### 3.1. Demographic and Clinical Characteristics

Of the 89 patients who underwent CEA, 31 (34.8%) had hyperuricemia. The demographic data of patients with and without hyperuricemia are presented in Table 1.

Demographic data were not significantly different between patients with and without hyperuricemia. The mean age of patients with and without hyperuricemia was 72.2 ± 9.2 years and 71.3 ± 8.4 years (*p* = 0.54), respectively. Among patients with hyperuricemia, 27 were male and 4 were female, whereas among patients without hyperuricemia, 51 were male and 7 were female (*p* = 0.92). The prevalence of stroke risk factors at baseline was high. Eighteen patients with hyperuricemia and thirty patients without hyperuricemia had dyslipidemia. In total, 90 (72.6%) patients had hypertension, 43 (34.7%) had diabetes mellitus, 15 (12.1%) had aortic disease, 5 (4.0%) had arteriosclerosis obliterans, 14 (11.3%) had chronic kidney disease, 7 (5.6%) had hemorrhagic stroke, and 70 (56.5%) had previous ischemic stroke.

Regarding medical therapy before CEA, 11 (35.5%) patients with hyperuricemia and 15 (25.9%) patients without hyperuricemia were prescribed antiplatelet agents. Two (6.5%) patients with hyperuricemia and five (8.6%) patients without hyperuricemia were prescribed anticoagulants. In total, 50 (40.3%) patients were taking statins, 9 (7.3%) were taking other lipid-lowering drugs, 85 (68.5%) were taking antihypertensive drugs, and 36 (29.0%) were taking antidiabetic drugs.

### 3.2. Gomori’s Methenamine Silver and HE Staining

The characteristics of the carotid plaques evaluated using HE staining revealed that type VI was the most frequent (n = 62; 69.6%), followed by type IV (n = 21; 23.6%) and type V (n = 6; 6.7%). Therefore, most of the participants in this study had complex plaques.

**Figure 1:**
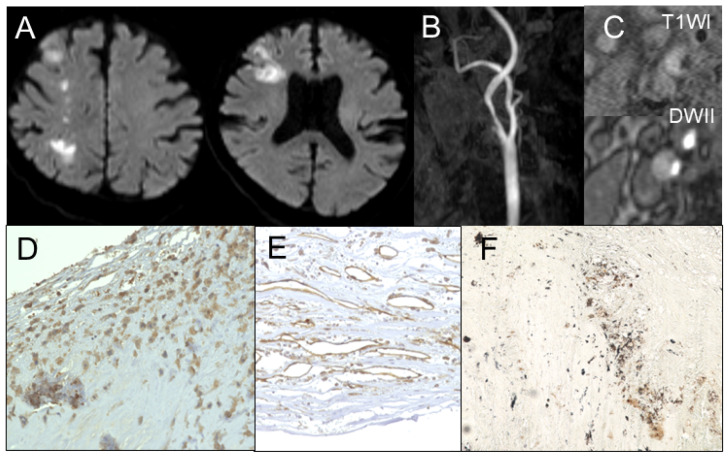
Illustrative case of unstable plaque. (**A**) Magnetic resonance imaging shows cerebral infarction in diffusion-weighted image. (**B**) Magnetic resonance angiography shows ipsilateral carotid stenosis. (**C**) Magnetic resonance imaging of plaque shows intraplaque hemorrhage. Immunohistochemistry shows densely expressed (**D**) CD68-positive cells and (**E**) CD31-positive microvessels. (**F**) Gomori’s methenamine silver staining reveals monosodium urate monohydrate deposits in shoulder lesions.

We performed Gomori’s methenamine silver and HE staining of CEA specimens from the 89 participants. In this cohort, 16 (51.6%) of the 31 patients with hyperuricemia showed positive results for Gomori’s methenamine silver staining. This positive result was observed for the shoulder lesion. In contrast, only 6 (10.3%) of 58 patients without hyperuricemia showed positive results for Gomori’s methenamine silver staining (Figure 1). These six patients were taking oral uric acid-lowering drugs. The LDL levels in patients with and without MSU deposits were 101.6 ± 32.0 mg/dL and 99.5 ± 38.6, respectively (*p* = 0.41). The corresponding CRP levels were 0.52 ± 0.31 and 0.41 ± 0.11, respectively (*p* < 0.01).

CD31-positive microvessels were observed in shoulder lesions. The number of CD31-positive microvessels was higher in patients with positive results for Gomori’s methenamine silver staining (128 ± 34.9 counts) than in those without positive results (55.8 ± 18.9 counts) (*p* < 0.01). Similarly, CD68-positive cells were expressed in the shoulder lesions. The number of CD68-positive microvessels was higher in patients with positive results for Gomori’s methenamine silver staining (156 ± 40.8 counts) than in those without positive results (50.1 ± 16.5 counts) (*p* < 0.01).

**Figure 2:**
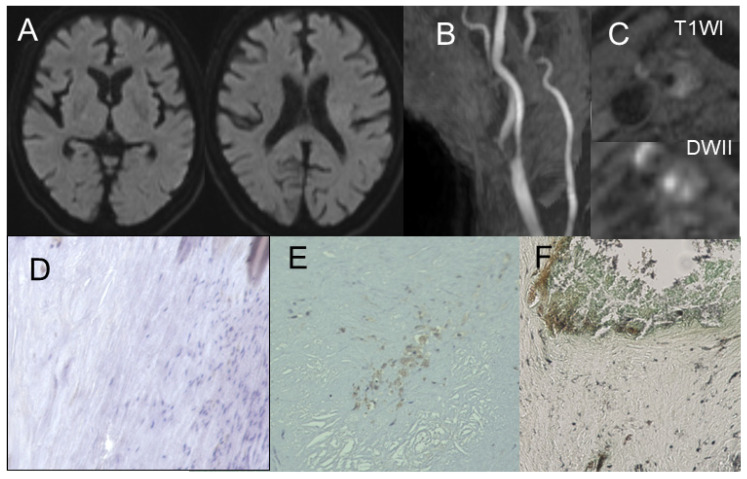
This figure illustrates a case of stable plaque. (**A**) Magnetic resonance imaging shows no cerebral infarction. (**B**) Magnetic resonance angiography shows ipsilateral carotid stenosis. (**C**) Magnetic resonance imaging of the plaque shows fibrous plaque. Immunohistochemistry shows the expression of a small number of (**D**) CD68-positive cells and (**E**) CD31-positive microvessels. (**F**) Gomori’s methenamine silver staining shows monosodium urate monohydrate deposits in shoulder lesions.

**Figure 3:**
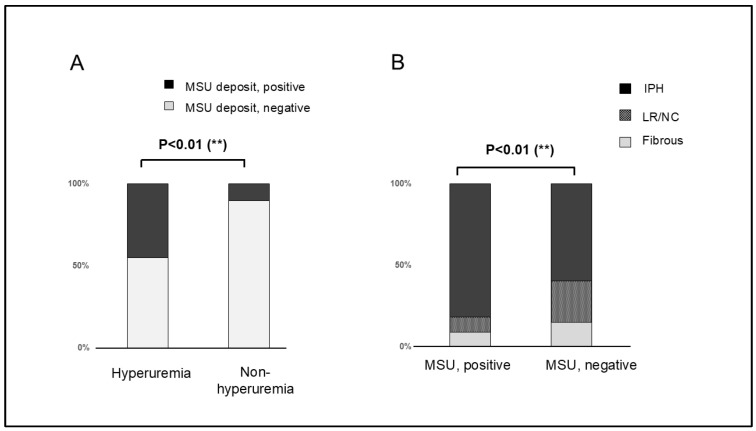
(**A**) Bar graph shows monosodium urate monohydrate (MSU) deposits in patients with and without hyperuricemia evaluated using Gomori’s methenamine silver staining. MSU deposits are more frequent in patients with hyperuricemia than in those without hyperuricemia (*p* < 0.01). (**B**) Intraplaque hemorrhage is more frequent in patients with MSU deposits than in those without MSU deposits (*p* < 0.01).

The serum urate acid level was 8.6 ± 2.2 mg/dL in patients with positive results for Gomori’s methenamine silver staining and 6.3 ± 1.9 mg/dL in patients with negative results for Gomori’s methenamine silver staining (*p* < 0.01). Illustrative cases of plaques with IPH (Figure 2) and fibrous plaques (Figure 3) are presented.

### 3.3. Correlation Between Radiological Findings and Plaque Pathology

Overall, 22 (24.7%) patients had MSU deposits and 67 (75.3%) patients did not. Plaque composition varied widely between patients with and without MSU deposits. The plaque composition in patients with MSU deposits included 18 plaques (81.8%) with IPH, 2 LR/NC (9.1%), and 2 fibrous plaques (9.1%). In contrast, plaque composition in patients without MSU deposits included 40 (59.7%) plaques with IPH, 17 (25.4%) LR/NC, and 10 (14.9%) fibrous plaques. Significant differences in plaque composition were observed between patients with and without MSU deposits (*p* < 0.01) (Figure 3).

Among the 31 patients with hyperuricemia, plaque composition varied widely between patients with and without MSU deposits. The plaque composition of the plaques with MSU deposits included 14 (87.5%) plaques with IPH, 1 (6.2%) LR/NC, and 1 (6.2%) fibrous plaque. In contrast, the plaque composition in the plaques without MSU crystal deposits included six (40.0%) plaques with IPH, three LR/NC (20.0%), and six (40.0%) fibrous plaques. Plaque composition was significantly different between plaques with and without MSU deposits (*p* = 0.01).

### 3.4. MSU Deposits Detected Using DECT and Its Accuracy

**Figure 4:**
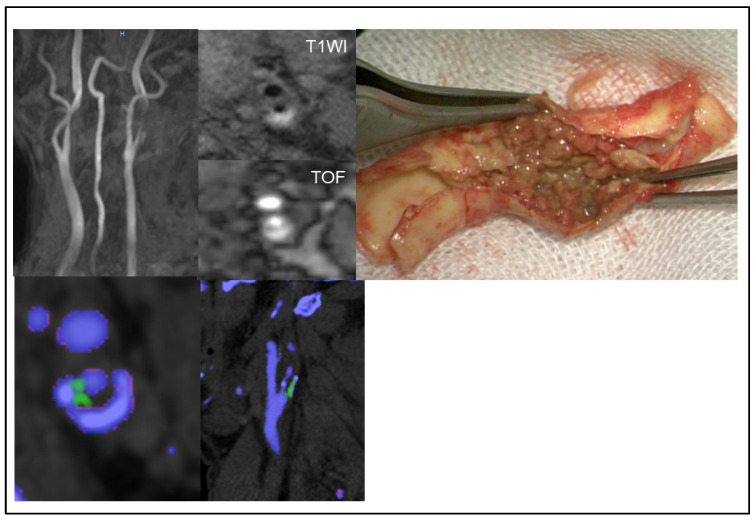
This figure illustrates a case of symptomatic carotid stenosis due to an unstable plaque. Dual-energy computed tomography shows monosodium urate monohydrate deposits as green spots.

MSU crystal deposits detected using DECT were observed in 13 patients (14.6%). An illustrative case of MSU crystal deposition in a carotid plaque is presented in Figure 4. However, the kappa value of the radiological findings of MSU detected using DECT and MSU crystal deposits detected using Gomori’s methenamine silver was poor (kappa = 0.34).

## 4. Discussion

This study has two main findings. First, most plaques expressing MSU deposits were plaques with IPH. Moreover, pathological findings indicated that most MSU crystal deposits were present in the shoulder lesions. Furthermore, CD31-positive microvessels and CD68-positive cells were expressed in the shoulder lesions. The number of CD31-positive microvessels and CD68-positive cells was higher in patients with MSU deposits than in those without MSU deposits. Therefore, MSU deposition may be related to the inflammation of carotid plaques. Second, the consistency of MSU crystal deposit identification between histopathology and DECT was poor (kappa = 0.34).

### 4.1. Correlation Between Plaque Composition, Inflammation, and MSU Crystal Deposition

Histopathological analysis showed that MSU crystal deposits were present in the shoulder lesions. Nardi et al. reported that MSU crystal deposits were expressed in atherosclerotic carotid plaques and were higher in patients with symptomatic carotid stenosis than in patients with asymptomatic carotid stenosis [12]. This suggests that uric acid deposits in carotid plaques may participate in the mechanism of stroke and that SUA levels may play a role as systemic biomarkers of cerebrovascular events. Further, these results indicate the existence of MSU deposits in shoulder lesions. The development of microvessels in the shoulder region, which triggers the migration of inflammatory cells, immune cells, and various cytokines, is a known pathological mechanism underlying the instability of carotid plaques. Similarly, our results revealed MSU deposits in shoulder lesions. These results support our finding that MSU crystal deposits may trigger inflammation and accelerate plaque instability. In the present study, type VI was the most frequent (n = 62; 69.6%), followed by type IV (n = 21; 23.6%) and type V (n = 6; 6.7%), showing that MSU deposits are largely found in complex carotid plaques. These findings support the role of MSU deposits as a possible mechanism underlying plaque instability.

Therefore, MSU crystals may serve as therapeutic targets for plaque stabilization. Yamamoto et al. reported that allopurinol, an inhibitor of xanthine oxidase activity, affected vascular remodeling and vascular smooth muscle cell proliferation in a spontaneously hypertensive rat model. These effects resulted in the inhibition of uric acid concentration in atherosclerotic plaque [13]. Kimura et al. suggested that the development of atherosclerosis and inflammation is promoted by uric acid in vivo. Lowering uric acid levels attenuates inflammation by activating the AMPK pathway. Their study provided evidence for the use of uric acid-lowering therapies for atherosclerosis [14]. Although this study revealed an association between MSU deposits and plaque composition, whether lipid lowering therapy can decrease the consequences of plaque vulnerability remains controversial. While uric acid is considered a potential therapeutic target, future clinical trials will be needed to confirm the efficacy of uric acid-lowering therapies for carotid plaque vulnerability.

It has also been suggested that inflammation related to MSU deposits in atherosclerotic plaques is mediated by the regulation of the hypoxia-inducible factor-1α pathway. We have previously reported that hypoxic conditions accelerate intraplaque neo-vessel formation [15]. These results suggest that hypoxia-related inflammation due to MSU crystal deposits may lead to IPH. For future studies, it is mandatory to determine the appropriate protocol to control SUA levels in patients at a high risk of stroke due to carotid plaques.

There are several possible methods through which MSU deposits in carotid plaques can be detected. Gomori’s methenamine silver staining is one such method. However, it is important to keep in mind that uric acid is quickly broken down once exposed to air. Pathological examination should be performed rapidly before degradation can occur. Electron microscopic observation is another known method but is unsuitable for preoperative testing. It is therefore desirable to visualize pathological MSU deposits using radiological methods.

### 4.2. MSU Crystal Deposits Detected Using DECT

In this study, the agreement between pathological MSU crystal deposits and MSU deposits detected using DECT was poor (kappa = 0.34). DECT is based on the principle that tissue attenuation depends on tissue density, chemical composition, and X-ray beam energy [16]. Recent studies have focused on whether DECT can provide evidence for cardiovascular MSU crystal deposition. However, the DECT-based identification of MSU deposition in the peripheral vasculature was initially considered artifactual [17,18]. We hypothesized that MSU deposits in cardiovascular vessel walls were not artifacts but true MSU deposits. However, our results did not support this hypothesis. The reason for the discrepancy between pathological MSU deposits and MSU deposits detected using DECT is that DECT may reflect noise within small structures in the carotid plaque, which may contribute to vascular artifacts. Moreover, the minimum size of MSU crystal deposits detected using DECT may be insufficient. DECT is an emerging clinical imaging technique; however, the current hardware and software may not be optimal for the long-term monitoring of MSU crystal deposits in carotid plaques. Artifacts can occur when using the DECT technique, and variations in threshold controls can substantially alter the urate signal. Overall, our results raised questions regarding the reliability of DECT as a diagnostic tool for MSU deposits and further underline the need for more extensive research to translate these techniques into practical, clinically reliable diagnostic tools.

### 4.3. Limitations

This study has several limitations. First, this was a single-center, retrospective study with a limited sample size. Therefore, the results of this study may have limited generalizability. Future studies should include a larger number of patients or a prospective multicenter cohort to address these limitations. Second, this was a cross-sectional study, and the causality of MSU deposits and inflammation could not be proven. A multicenter prospective cohort study to acquire longitudinal data is required to resolve these issues. Third, the cross-sectional design meant that it was not possible to confirm that uric acid-lowering drugs directly prevent MSU deposition in carotid plaques. Future prospective longitudinal studies are therefore warranted. Finally, quantitative pathological assessment is required to evaluate MSU deposits in carotid plaque, because uric acid is quickly broken down once exposed to air.

## 5. Conclusions

In conclusion, most plaques expressing MSU were plaques with IPH. Furthermore, pathological findings indicated that most MSU crystal deposits were present in the shoulder lesions. Moreover, CD31-positive microvessels and CD68-positive cells were observed in the shoulder lesions. The number of CD31-positive microvessels and CD68-positive cells was higher in patients with MSU deposits than in those without MSU deposits. MSU deposition may be related to the inflammation of carotid plaques.

## Figures and Tables

**Table 1:** The demographic data of patients with and without hyperuricemia.

	With Hyperuricemia	Without Hyperuricemia	*p* Value
Number	n = 31 (34.8%)	n = 58 (65.2%)	
**Age (years)**	72.2 ± 9.2	71.3 ± 8.4	0.54
**Gender**			
Male	27	51	0.92
Female	4	7	
**Medical comorbidity**			
Hypertension	25	46	0.48
Diabetetes	15	27	0.52
Hypercholesterolemia	18	30	0.27
Coronary artery disease	12	18	0.49
Aortic disease	6	10	0.78
ASO	5	9	0.98
Chronic renal failure	12	18	0.45
**History of stroke**			
Ischemic stroke	8	13	0.79
Ipsilateral ischemic stroke	5	8	0.76
Contra- or posterior circulation	3	5	0.98
Hemorrhagic stroke	2	4	0.95
**Medical therapy prior to CEA**			
Hyperuricemia	25	12	<0.01
HT	24	42	0.8
HL statin	18	26	0.27
HL other	5	7	0.74
DM	13	20	0.49
Antiplatelet	11	15	0.81
Anticoaglunt	2	5	0.95
**Radiological findings**			
Degree of stenosis(%)	71.0 ± 11.3	69.5 ± 11.3	0.48
Plaque unceration presence	17	22	0.19
Ipsilateral intracranial atherosclerosis	5	11	0.97
Plaque composition			
Fibrous	2	4	0.86
LR/NC	8	11	
IPH	21	43	

## Data Availability

The data analyzed in the current study are available from the corresponding author upon reasonable request.
